# Severe Phenotype of APECED (APS1) Increases Risk for Structural Bone Alterations

**DOI:** 10.3389/fendo.2020.00109

**Published:** 2020-03-06

**Authors:** Saila Laakso, Joonatan Borchers, Sanna Toiviainen-Salo, Minna Pekkinen, Outi Mäkitie

**Affiliations:** ^1^Children's Hospital, Pediatric Research Center, University of Helsinki and Helsinki University Hospital, Helsinki, Finland; ^2^Folkhälsan Research Center, Helsinki, Finland; ^3^Research Program for Clinical and Molecular Metabolism, Faculty of Medicine, University of Helsinki, Helsinki, Finland; ^4^Department of Pediatric Radiology, Medical Imaging Center, Helsinki University Hospital, Helsinki, Finland; ^5^Department of Molecular Medicine and Surgery, Karolinska Institutet, and Clinical Genetics, Karolinska University Hospital, Stockholm, Sweden

**Keywords:** autoimmune polyendocrinopathy syndrome type 1, bone, hypoparathyroidism, adrenal insufficiency, bone density, child

## Abstract

**Objective:** Immunological abnormalities, the resulting endocrinopathies and their treatments may impact bone health in patients with autoimmune polyendocrinopathy–candidiasis–ectodermal dystrophy (APECED, APS1). The aim of the present study was to describe skeletal characteristics in patients with APECED and the prevalence and risk factors of compromised bone health.

**Patients and methods:** We performed a cross-sectional study on 44 patients (27 females) with APECED and 82 age-, gender- and ethnicity-matched control subjects (54 females). We determined the prevalence of osteoporosis by dual-energy X-ray absorptiometry and skeletal characteristics by peripheral quantitative computed tomography at radius and tibia.

**Results:** Patients were examined at the median age of 37.8 years (range, 7.0–70.1). Dual-energy X-ray absorptiometry indicated osteoporosis in four adult patients (9%); radiographs showed vertebral fractures in three patients. The prevalence of multiple non-spinal fractures was higher in patients than in controls. On peripheral quantitative computed tomography, bone characteristics at distal and proximal radius did not differ between the groups. At distal tibia, patients had lower total (*p* = 0.009) and trabecular (*p* = 0.033) volumetric bone mineral density. At the proximal tibia, patients had lower cortical thickness (*p* < 0.001) than controls. Severity of APECED phenotype influenced both radial and tibial characteristics: cortical thickness and total and trabecular volumetric bone mineral density were lower in patients with ≥7 disease manifestations as compared with more mildly affected patients, whose values were similar to controls.

**Conclusions:** APECED associated with bone structural alterations, especially in patients with a high number of disease manifestations. This may increase the risk of fractures with aging, but symptomatic osteoporosis was rare.

## Introduction

Autoimmune polyendocrinopathy-candidiasis-ectodermal dystrophy (APECED; OMIM 240300) or autoimmune polyglandular syndrome type 1 (APS1) is an autosomal-recessive disorder caused by mutations in the autoimmune regulator (*AIRE*) gene. Based on murine studies, Aire is known to induce central tolerance by promoting the expression of peripheral tissue-restricted autoantigens to maturing T cells in the medullary epithelial cells of the thymus. (1, 2) In APECED, the negative selection of autoreactive T cells is disturbed, leading to insufficient suppression of autoimmunity (3). Parathyroid, adrenal, and gonadal insufficiencies are the most common endocrinopathies which usually manifest in childhood or young adulthood, but new manifestations appear throughout life. The clinical course and sequence of manifestations in APECED was initially characterized in Finland, where the disease is more frequent than elsewhere due to a founder mutation (4).

The immunological abnormalities, the resulting endocrinopathies and their treatments may impact bone health. We previously showed in a smaller cohort of patients with APECED that osteopenia and osteoporosis are prevalent, as evaluated by DXA (5). These observations prompted us to carry out a more extensive case-control study involving 44 patients with APECED and 82 matched controls to describe bone health characteristics using in addition to DXA also peripheral quantitative computed tomography (pQCT), which provides more detailed information on bone size and volumetric bone mineral density (vBMD) in trabecular and cortical bone compartments.

## Methods

### Subjects

We invited to the study all patients who have participated previous large Finnish studies on APECED (6). Additional patients were recruited from other university hospitals and central hospitals throughout the country by contacting the respective endocrine and pediatric endocrine units. Further, patients were recruited through the patient organization. Of the 91 Finnish patients enrolled in previous studies (6), 61 were alive at the time of recruitment. In addition, four new pediatric patients have been diagnosed during the past 10 years. Of the total of 65 surviving patients, 44 (68%) consented and were included in the present study.

Adult control subjects were identified through population register and invited by letters to participate. We selected age-, gender- and ethnicity-matched healthy controls for each patient with APECED; subjects with any daily medication affecting bone health, immunity or metabolism were excluded. We initially invited 4–16 matched control subjects for each adult patient and then enrolled to the study 1 or 2 of those who first responded positively to the invitation. Seven control subjects were excluded because of daily medication affecting bone health, immunity, or metabolism. For the 7 APECED patients who were 7–16.5 years old we selected historical controls from a cohort of healthy school children who participated in 2007–2008 a study on bone health (7). An ethical approval was obtained from the Research Ethics Committee of the Hospital District of Helsinki and Uusimaa. Informed written consent was obtained from all study participants or their guardians (subjects aged <18 years).

### Clinical Assessments

For the patients with APECED, clinical details were collected from hospital records, by patient interview and with a questionnaire inquiring medical history, fractures, medications, skeletal symptoms, and life-style factors such as smoking, alcohol use, and current total physical activity. Ages at the diagnosis of disease manifestations were collected from hospital records: chronic mucocutaneous candidiasis, primary adrenal insufficiency (PAI), hypoparathyroidism, primary ovarian insufficiency (POI)/hypogonadism, hypothyroidism, growth hormone deficiency, diabetes, gastritis, malabsorption, hepatitis, asplenia/hyposplenia, nephritis, keratitis, alopecia, vitiligo, enemal dysplasia, rash with fever, azospermia, and transient metaphyseal dysplasia. Malabsorption was defined as chronic diarrhea or exocrine pancreas failure. Postmenopausal state was determined either by amenorrhea after age 40 years or discontinuation of hormone replacement therapy in those with POI. Smoking history was recorded positive, if the subject had smoked at least 100 cigarettes during life-time. Physical activity (min/day) consisted of 12 months' retrospective recall of activity related to travel to school or work and supervised or independent leisure-time activity. Clinical evaluation included anthropometry and assessment of pubertal stage in children. Weight was measured in light clothing with a Seca digital scale to the nearest 0.1 kg. Height was measured to the nearest 0.1 cm with a fast stadiometer connected to the scale. Height and BMI Z-scores were calculated according to Finnish growth standards (8).

Adult control subjects were clinically assessed in the same way as patients, and they were similarly inquired about fractures, medications, skeletal symptoms, and life-style factors such as smoking, alcohol use, and current total physical activity. For the control children, the weights and heights were measured in the same way as for patients, but their pubertal staging was determined by questionnaires and hormonal measurements, as previously reported (7).

### Genetic Analyses

All *AIRE* (NM_000383.4) pathogenic variants: c.769C>T, p.Arg257Ter; c.967_979del13, p.Leu323fs; c.932G>A (p.Cys311Tyr); and c.891C>A (p.Asp297Glu) were detected by Sanger sequencing using standard protocols. Primer sequences are available from the authors upon request.

### Imaging Studies

In the patient group, bone mineral density (BMD) (for lumbar spine, total hip, and whole body) was determined by DXA (Lunar Prodigy, enCore DPX-NT, General Electric Company, Freiburg, Germany). Scans were performed by certified technicians. Calibration was performed with a spine phantom. Reducibility of whole body DXA measurement is for BMD, 0.85%, for bone mineral content, 0.45%, and for bone area, 0.78% (9). In children, BMD Z-scores were calculated according to bone age; BMD T-scores were used in adult patients. Equipment-specific reference data were used to determine Z- and T-scores. Spinal compression fractures were determined from lateral spinal radiographs as vertebral body height reduction exceeding 20% in relation to posterior height or adjacent vertebral bodies (10). In children and adolescents (<18 years of age) with APECED, a hand radiograph was obtained to evaluate skeletal maturity (11). In the control group, no DXA measurement or X-ray imaging was performed.

In both patients and controls, we used pQCT (XCT-2000 scanner, Stratec Medizintechnik GmbH, Pforzheim, Germany) to evaluate bone characteristics in non-dominant radius (4 and 66% sites) and tibia (4%, 38%). Volumetric BMD and geometry in the total, trabecular and cortical bone compartments were determined. Scans were performed by one trained and experienced operator. The repeatability of the pQCT was evaluated with daily measurements of phantom provided by the manufacturer. For total, trabecular and cortical cross-sectional area and density CV%s were 0.24 and 0.27; 0.25 and 0.34; and 0.25 and 0.31, respectively. In the control children, pQCT scans were performed only at the radial sites. Values were compared between the patients and controls and further correlated with disease features in the patients.

### Biochemical Measurements

Blood samples and second void urine were collected between 07:00 and 10:00 h after an 8–12-h fast in both patients and control subjects. Plasma calcium, ionized calcium, phosphate, alkaline phosphatase (ALP), and creatine were measured using standard methods at the University Hospital's core laboratory HUSLab. Plasma 25-hydroxy-vitamin D [25(OH)D] was quantified with the electrochemiluminescence immunoassay (ECLIA, Roche Diagnostics) or a chemiluminescent microparticle immunoassay (CMIA, Abbott Laboratories) except in the control children whose 25(OH)D was measured assayed by high-performance liquid chromatography (HPLC) (7). Serum IGF-1 was quantified with the immunochemiluminometric assay on the IMMULITE 2000XPi analyzer (Siemens, Llanberis, UK). Detection limit of the assay was 3 nmol/L, and inter-assay coefficient of variation was <5% in the range 12–120 nmol/L. Serum procollagen type 1 aminoterminal propeptid (PINP) was measured with immunochemiluminometric assay [CLIA, IDS-iSYS (IS-4000), Immunodiagnosticsystems] or with ECLIA (Elecsys total P1NP for cobas e801, Roche Diagnostics). Second void urine samples were collected and analyzed for urinary calcium, phosphate and creatinine using standard methods. Urinary collagen-1 aminoterminal telopeptide (INTP) was measured with enzyme-immunologic method (OSTEOMARK NTx Urine Elisa, Alere).

### Statistics

Results are reported as median (range) or mean (95% confidence interval). Kolmogorov-Smirnov test was used to test for normality of distributions. Differences between two groups were tested with Mann-Whitney *U*-test and Student's t test, as appropriate. Differences between three groups were tested with Kruskall-Wallis non-parametric test. Chi-square test and Fischer's exact test were applied for the categorical variables, as appropriate. Significance level was determined as *P* < 0.05. All the statistical analyses were performed with SPSS Statistical package (version 24.0.0.2) and GraphPad Prism (version 8.2.0).

## Results

### Cohort Characteristics

Altogether 44 patients (27 females) with APECED were examined at the median age of 37.8 years (range, 7.0–70.1 years). Seven patients were below 18 years of age. Characteristics of the patients are presented in Table 1. All patients harbored at least one copy of the Finnish founder mutation c.769C>T, p.Arg257Ter in the *AIRE* gene (NM_000383.4). Altogether 77% were homozygous for this pathogenic variant while in the others it was compounded with c.967_979del13 (p.Leu323fs) (*n* = 5), c.932G>A (p.Cys311Tyr) (*n* = 2), or c.891C>A (p.Asp297Glu), (*n* = 2).

**Table 1:** Characteristics of the 44 Finnish patients with APECED.

**Characteristic**	**Number (%)**	**Age at onset (years) Median (range)**
Median age (years, range)	37.8 (7.0–70.1)	
Subjects younger than 18 years	7 (16%)	
Female gender n (%)	27 (61%)	
**Genotype**		
c.769C>T/c.769C>T	34 (77%)	
c.769C>T/c.967_979del13	5 (11%)	
c.769C>T/other	5 (11%)	
**Manifestations**		
Hypoparathyroidism	37 (84%)	5.8 (1.6–42.4)
Adrenocortical insufficiency	36 (82%)	10.0 (2.5–32.9)
Hypogonadism	20 (45%)	16.0 (11.4–36.5)
Growth hormone deficiency	5 (11%)	10.5 (5.9–15.1)
Exocrine pancreatic failure	4 (9%)	19.7 (4.4–41.7)
Tubulointerstitial nephritis	4 (9%)	15.6 (9.5–20.6)
**Medications**		**Median dose per day**
Calcium supplement (mg/d)	36 (82%)	1000 (500–3000)
Alfacalsidol (μg/d)	20 (51%)	1.8 (0.5–10)
Dihydrotachysterol (μg/d)	16 (41%)	750 (360–3000)
Vitamin D_3_ (μg/d)	24 (55%)	11 (5–170)
Hydrocortisone (mg/d)	33 (75%)	20 (10–30)
DHEA (mg/d)	6 (14%)	16 (6–50)

The median number of disease manifestations in patients was 6 (range, 1–12). Hypoparathyroidism had been diagnosed in 84% of the patients at the median age of 5.8 years (Table 1). PAI had been diagnosed in over 80% of the patients at the median age of 10.0 years; 75% of them received short-acting hydrocortisone replacement therapy while 25% received other cortisone medications alone (prednisone, *n* = 2; prednisolone, *n* = 1) or in combination with hydrocortisone (prednisolone, *n* = 1). Median daily hydrocortisone equivalent dose was 0.32 mg/kg (range, 0.16–0.53). POI had been diagnosed in 67% of the females at the age of 11.4–36.5 years. Seven females (26%) were postmenopausal. Two male patients were on testosterone therapy due to primary hypogonadism diagnosed at 15 and 21 years. Tubulointerstitial nephritis had been diagnosed in four patients.

Altogether 82 age-, gender- and ethnicity-matched control subjects were examined. The median age of the 68 adult control subjects (65% females) was 46 years. Thirteen of the 44 females (30%) were postmenopausal. The age range of the 14 control children (71% females) was 7.4–16.5 years (Table 2).

**Table 2:** Characteristics in the pediatric and adult patients with APECED in comparison with age- and gender-matched control subjects.

	**Patients**	**Controls**	
**Median (range)**	***N* = 7**	***N* = 14**	***P* value**
CHILDREN			
Gender (female/male, n)	5/2	10/4	
Age (years)	12.5 (7.0–16.5)	12.5 (7.4–16.5)	0.8
Bone age (years)	9.6 (5.3–16.5)		
Pubertal stage (prepubertal/pubertal!!!break!!!/postpubertal, n)	4/2/1	8/3/3	> 0.9
Height (cm)	152 (116–166)	154 (125–167)	0.5
Height Z-scores	−1.6 (−3.1–+0.8)	−0.2 (−1.1–+0.9)	0.016
Weight (kg)	42.1 (17.6–60.6)	43.5 (21.6–85.9)	0.9
BMI Z-scores	+0.1 (−2.3–+1.4)	−0.3 (−1.8–+2.7)	0.9
**Mean (95% CI)**	***N*** **=** **37**	***N*** **=** **68**	***P*** **value**
ADULTS			
Age (years)	42.8 (38.6–47.1)	46.0 (43.0–49.1)	0.2
Female	22 (60%)	44 (65%)	0.7
Postmenopausal women*	7 (19%)	13 (19%)	> 0.9
Duration of HP [years, *n* = 31, median (range)]	32.5 (1.6–57.8)		
Duration of PAI [years, *n* = 30, median (range)]	30.4 (13.7–49.5)		
Height SDS	−1.0 (−1.4−0.7)	−0.2 (−0.4–+0.1)	<0.001
BMI (kg/m^2^)	23.1 (21.3–24.8)	25.7 (24.6–26.9)	0.007
Smoking (n)	17 (46%)	30 (44%)	0.9
Alcohol use (doses per 2 wk)	8.9 (4.2–13.6)	8.4 (5.7–11.0)	0.8
Physical activity (min/day)	57 (30–84)	51 (44–59)	0.7
Calcium from dairy products (mg/day)	557 (423–692)	358 (295–422)	0.009
Total calcium intake (mg/day)^+^	1600 (1330–1870)	390 (318–462)	<0.001
Osteoporosis in 1^st^ degree relative (n)	5 (14%)	14 (21%)	0.059

HP, hypoparathyroidism; PAI, primary adrenal insufficiency. *, patients with APECED were regarded as postmenopausal if they had experienced menopause at age of over 40 years or hormone replacement therapy on primary ovarian insufficiency had been stopped. ^+^, total calcium intake includes calcium from dairy products and calcium supplements.

### Anthropometry and Life-Style Factors Affecting Bone Health

The characteristics of the patients with APECED and their matched control subjects are described in Table 2. Seven children and adolescents with APECED were examined at the median age of 12.5 years. In five of them, bone age was delayed by more than a year from calendar age, and their DXA-derived BMD Z-scores were calculated according to bone age. Their pubertal stage, and median height and BMI did not differ from the age- and gender-matched control subjects, but their height Z-scores were lower than in control subjects (Table 2).

Altogether 37 adult patients with APECED were examined at median age of 42.8 years and their data were compared with 68 age- and gender-matched control subjects (Table 2). The patients were shorter, and their BMI was lower than in the control subjects. Patients used more calcium supplements and dairy products than control subjects. Lifestyle characteristics including smoking, alcohol use, and physical activity did not differ between the groups. No significant differences in the hereditary risk of osteoporosis, as evaluated by family history for osteoporosis, was seen between the patients and control subjects (Table 2).

### Fractures

Three patients (7%) had 1–3 vertebral fractures on spinal radiographs. Mild anterior wedge compression fractures (vertebral height reduction 21–30%) were observed in the lower thoracic spine in the three female patients, of whom one had had acute back pain at the time of vertebral fracture. Regarding non-spinal fractures, altogether 18 patients had a positive fracture history (1–5 fractures). Of the fractures, 44% involved upper extremity, 27% lower extremity, and 29% other bones including clavicle, jaw, rib, and coccyx. Of the total of 34 fractures, 41% were moderate- to high-energy fractures, and two of them had required surgical treatment. The proportion of subjects who had experienced at least one fracture did not differ between the patients and the 82 matched control subjects [18(41%) vs. 25(31%), *p* = 0.3]. However, the proportion of subjects who had sustained multiple fractures was higher in patients than controls: 23% of patients (*n* = 10) and 9% of the controls (*n* = 7) had experienced two or more fractures (*p* = 0.033).

### DXA Measurements and Risk of Osteoporosis

In the patients with APECED, the median BMD Z/T-scores were +0.7 (range, −3.5–+3.4) for whole body, +0.9 (−3.3–+3.9) for lumbar spine, and +0.7 (−2.9–+3.3) for femoral neck. Osteoporosis (*n* = 4) or osteopenia (*n* = 6) was evident in 10 patients (23%). Altogether four adult patients (9%, one female) had either whole body or lumbar spine BMD T-score below −2.5 at the age of 23.1 to 44.1 years. Three of them had PAI, three had hypoparathyroidism, and two had hypogonadism. In addition, three of them had received growth hormone therapy due to growth hormone deficiency. Two of them had diagnosis of exocrine pancreas failure, but all four had chronic diarrhea. One patient had stable tubulointerstitial nephritis. One patient had received bisphosphonate treatment from age 58 to 66 years. The treatment had been discontinued 4 years prior the study evaluation and his current lumbar spine BMD T-score was −2.3 and within the osteopenic range. On the other hand, in the patient cohort, ten of the 37 patients with hypoparathyroidism (27%) had BMD Z/T-scores above +2.5 at one or several measurement sites, whereas none of the patients without hypoparathyroidism had high BMD.

### Bone Characteristics Evaluated by pQCT

**Figure 1:**
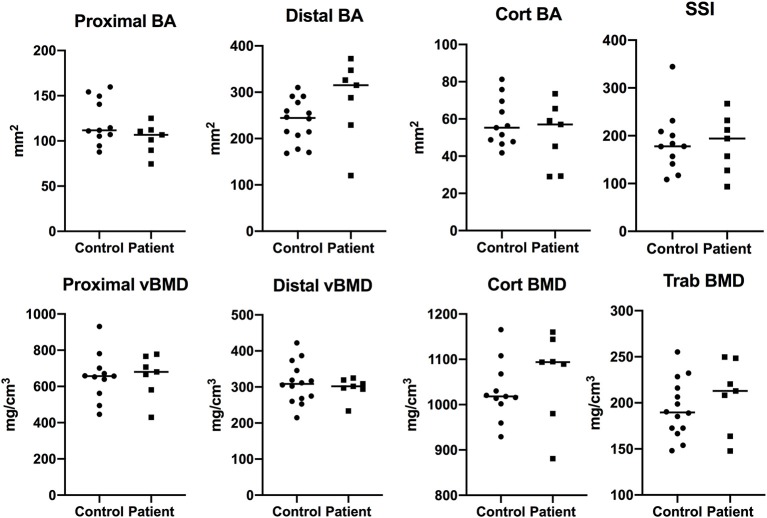
Bone characteristics at radial site in children with APECED (*n* = 7) in comparison to age- and gender-matched control children (*n* = 14). Total and cortical bone area (BA), total, cortical, and trabecular volumetric bone mineral density (vBMD), and strength-strain indices measured at proximal (66%) and distal (4%) site of radius with pQCT.

**Figure 2:**
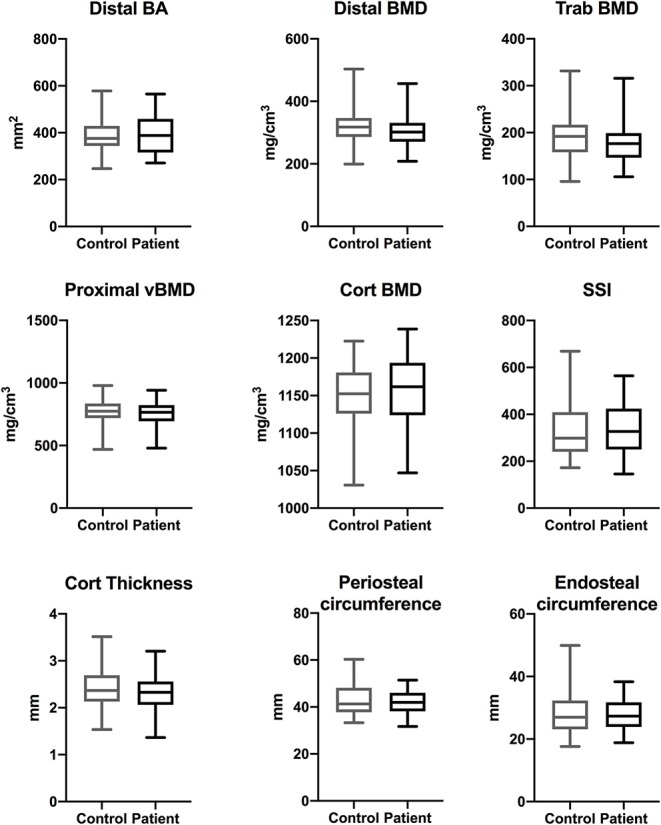
Bone characteristics at radial site in adults with APECED (*n* = 37) in comparison to age- and gender-matched control subjects (*n* = 68). Total bone area (BA), total, cortical and trabecular volumetric bone mineral density (vBMD), strength-strain indices, and cortical dimensions measured at proximal (66%) and distal (4%) site of radius with pQCT.

In order to evaluate bone properties more in detail in peripheral sites, we performed pQCT measurement on all the patients and control subjects. At the radial sites (distal 4%, proximal 66%), which reflect mainly bone metabolism without major effects of bone loading, we saw no significant differences in bone area, trabecular, cortical or total vBMD, or strength-strain-index between the patients and controls either in childhood (Figure 1) or adulthood (Figure 2).

**Figure 3:**
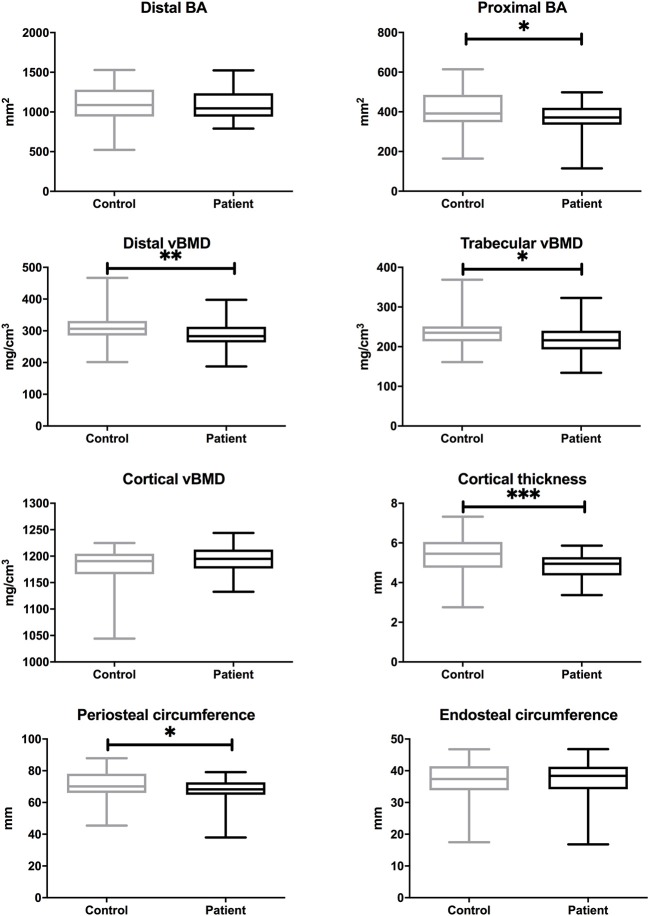
Bone characteristics at tibial site in adults with APECED (*n* = 37) in comparison to age- and gender-matched control subjects (*n* = 68). Total bone area (BA), total, cortical and trabecular volumetric bone mineral density (vBMD), and cortical dimensions measured at proximal (38%) and distal (4%) site of tibia with pQCT. *P* values are indicated with asterisks: *, < 0.05; **, < 0.01; ***, < 0.001.

In order to evaluate the bone properties on the site that is under the influence of weight bearing and physical activity, we assessed bone characteristics at the tibial sites (distal 4%, proximal 38%); data were available only for the adult subjects. Total bone area was smaller in the patients at the proximal site [mean difference (95% CI), −40.0 (−73.6−6.4) mm^2^, *p* = 0.020], but did not differ between the groups at the distal site (Figure 3). At the distal site, patients had lower total vBMD [−24.2 (−42.2−6.3) mg/cm^3^, *p* = 0.009] and trabecular vBMD [−17.6 (−33.8−1.5) mg/cm^3^, *p* = 0.033]. At proximal site, patients had lower cortical thickness [−0.61 (−0.94−0.27) mm, *p* < 0.001] as compared with controls. Further, the periosteal circumference was smaller in patients [−3.5 (−6.9−0.1) mm, *p* = 0.042], but endosteal circumference did not differ between the groups, in agreement with the smaller cortical thickness seen in patients (Figure 3).

**Figure 4:**
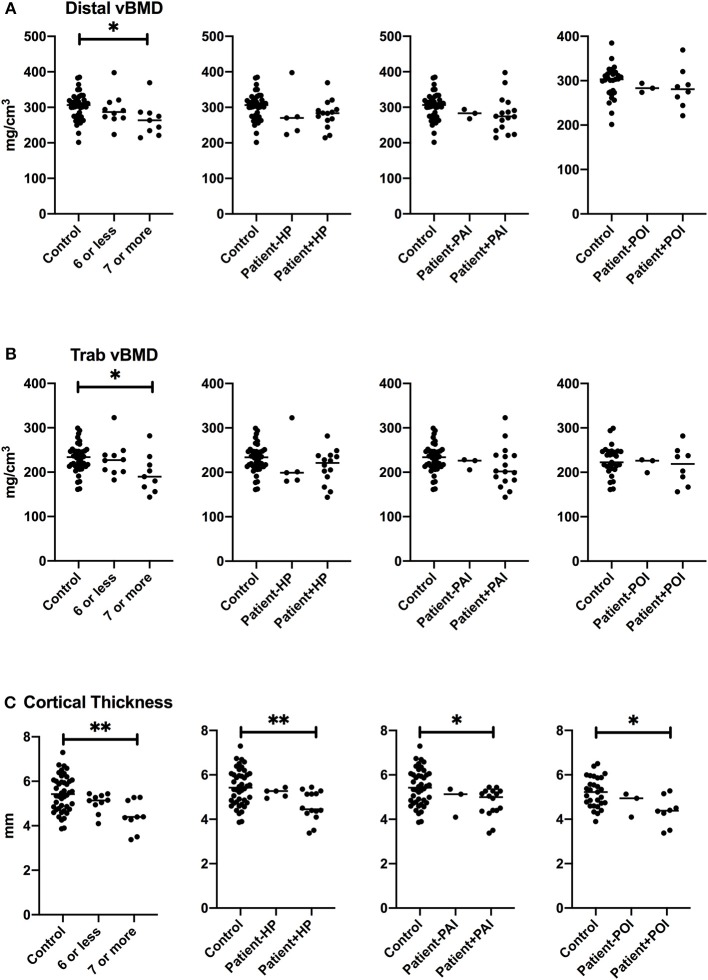
Bone characteristics at tibial site in patients older than 40 years with specific manifestation of APECED. Total volumetric bone mineral density at distal site **(A)**, trabecular volumetric bone mineral density **(B)**, and cortical thickness **(C)** in the patients with ≥7 disease manifestations, hypoparathyroidism (HP), primary adrenal insufficiency (PAI), or primary adrenal insufficiency (POI) in comparison to other patients and age- and gender-matched control subjects. *P* values are indicated with asterisks: *, < 0.05; **, < 0.01.

Aging may potentiate the effects of risk factors for impaired bone health, and disease severity may influence skeletal consequences of APECED. We therefore evaluated whether greater severity of APECED associated with lower total or trabecular vBMD at distal tibial site or with lower cortical thickness at proximal tibial site at older age. We performed a sub-group analysis in subjects older than 40 years and compared patients with 7 or more disease manifestations (*n* = 10) with patients with six or less disease manifestations (*n* = 11), and with age-and gender-matched control subjects (Figure 4). All three bone parameters—total vBMD and trabecular vBMD at the distal tibia, and cortical thickness at the proximal tibia, were lower in patients with more severe phenotype of APECED (more disease components), whereas no significant differences in the parameters were observed between patients with a milder phenotype and control subjects. When we performed the analyses separately for men and women, the more severe phenotype associated with lower trabecular vBMD at the tibial site in male patients (≥7 manifestations, *n* = 7) and with smaller cortical thickness at the tibial site in female patients (≥7 manifestations, *n* = 11, data not shown).

In order to evaluate how hypoparathyroidism, PAI, and POI contributed to tibial bone impairment, we divided the patients older than 40 years into groups by a specific disease component. Smaller cortical thickness associated with all these three disease manifestations, but no significant difference in total vBMD or trabecular vBMD at tibial site between the patients with hypoparathyroidim, PAI or POI was observed (Figure 4).

**Figure 5:**
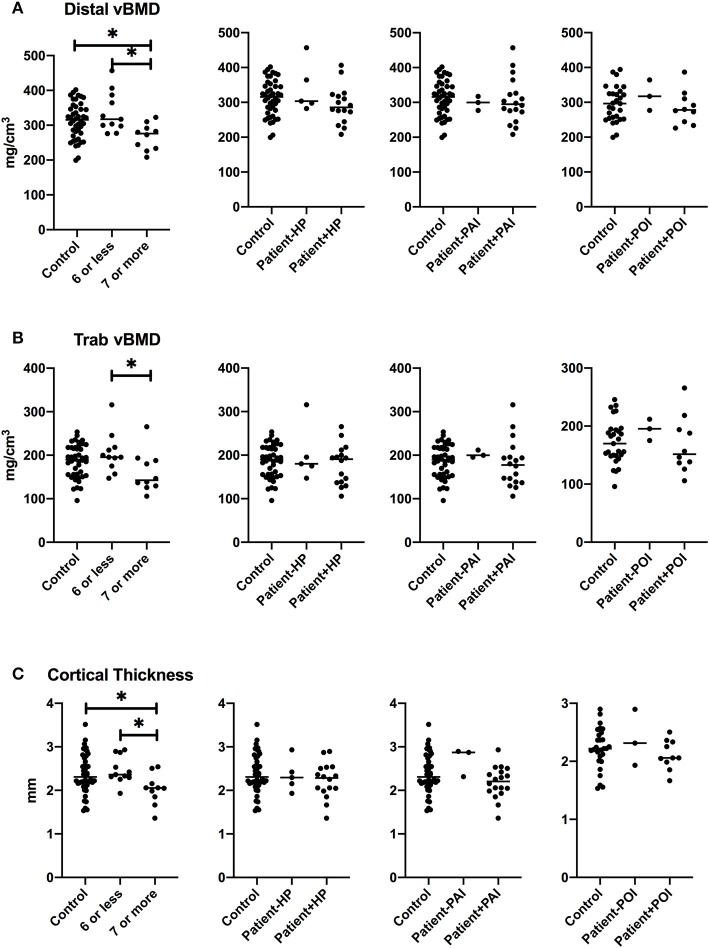
Bone characteristics at radial site in patients older than 40 years with specific manifestation of APECED. Total volumetric bone mineral density at distal site **(A)**, trabecular volumetric bone mineral density **(B)**, and cortical thickness **(C)** in the patients with ≥7 disease manifestations, hypoparathyroidism (HP), primary adrenal insufficiency (PAI), or primary adrenal insufficiency (POI) in comparison to other patients and age- and gender-matched control subjects. *P* values are indicated with asterisk: *, < 0.05.

On the radial site, cortical thickness, total vBMD, and trabecular vBMD were similarly lower in patients with more severe phenotype of APECED (≥7 disease components) as compared with more mildly affected patients or the matched controls, but no significant difference was observed when the groups were compared according to specific disease components—hypoparathyroidism, PAI, or POI (Figure 5).

When we analyzed pQCT parameters of radial and tibial sites separately among the female adult patients with APECED, postmenopausal state associated with lower cortical vBMD at radial site and larger endosteal circumference at both radial and tibial site (data not shown).

### Bone Metabolism

When we compared biochemical markers of bone metabolism, patients younger than 18 years, of whom 6 had hypoparathyroidism, had lower serum calcium concentration and higher plasma 25(OH)D than control subjects (Table 3). All pediatric patients had sufficient (>50 nmol/L) plasma 25(OH)D levels and no difference in the concentrations of the bone formation marker PINP was seen between the groups (Table 3). Adult patients had lower serum ionized calcium concentration, higher plasma phosphate concentration, and higher urinary calcium secretion reflecting treated hypoparathyroidism. Mean serum creatine was higher in the adult patients than controls. Plasma 25(OH)D, ALP, PINP and urinary INTP concentrations did not differ between the adult groups (Table 3). Plasma 25(OH)D was below 50 nmol/l in 24% of the patients (*n* = 9) in comparison to 9% of the control subjects (*n* = 6, *p* = 0.032).

**Table 3:** Biochemical parameters in pediatric and adult patients with APECED in comparison to control subjects.

**Characteristic Median (range)**	**Patients**	**Control subjects**	***P* value**
**Children**	***N* = 7**	***N* = 14**	
S-Ca (mmol/L)	2.23 (1.9–2.4)	2.38 (2.3–2.5)	0.022
P-Pi (mmol/L)	1.71 (1.25–1.84)	1.43 (1.12–1.68)	0.068
P-ALP (U/L)	134 (38–227)	197 (50–438)	0.4
P-25(OH)D (nmol/L)	79 (53–113)	39 (30–60)	<0.001
IGF-1 (nmol/L)	23 (10-71)		
S-PINP (μg/L)	389 (10–838)	608 (106–904)	0.4
U-INTP (nmol/mmol crea)	270 (30–590)		
U-Ca to U-Crea ratio	0.52 (0.07–1.31)	0.21 (0.02–0.41)	0.083
U-Pi to U-Crea ratio	1.92 (0.88–2.65)	1.32 (0.69–2.51)	0.4
**Adults**	**Patients**	**Controls**	***P*** **value**
**Mean (95% CI)**	***N*** **=** **37**	***N*** **=** **68**	
S-Ca-ion (mmol/L)	1.08 (1.03–1.13)	1.20 (1.19–1.20)	<0.001
P-Pi (mmol/L)	1.36 (1.25–1.46)	0.98 (0.95–1.01)	<0.001
P-ALP (U/L)	61 (53–70)	67 (62–72)	0.3
P-25(OH)D (nmol/L)	74 (65–82)	81 (75–88)	0.2
P-crea (umol/L)	87 (79–94)	73 (70–76)	0.001
S-PINP (μg/L)	56 (43–70)	53 (48–59)	0.7
U-INTP (nmol/mmol crea)	44 (28–59)	42 (37–47)	0.8
U-Ca to U-Crea ratio	0.40 (0.30–0.51)	0.21 (0.19–0.24)	<0.001
U-Pi to U-Crea ratio	1.88 (1.41–2.34)	1.62 (1.46–1.77)	0.3

*S, serum; Ca, calcium: Pi, phosphate; ALP, alkaline phosphatase; 25(OH)D, 25-hydroxy vitamin D; PINP, procollagen type 1 aminoterminal propeptid; U, urinary; INTP, collagen-1 aminoterminal telopeptide; crea, creatine*.

## Discussion

This study evaluated in detail the skeletal characteristics, and the prevalence of and risk factors for compromised bone health in patients with APECED. Evaluations with pQCT revealed that adult patients had lower total and trabecular vBMD at the distal site of tibia, and lower cortical thickness at proximal site when compared with age- and gender-matched controls. The differences in bone structure and bone metabolism reflected both PAI and hypoparathyroidism, as well as the more severe phenotype of APECED. However, the skeletal characteristics were surprisingly good, and osteoporosis, based on DXA measurements, was relatively rare in patients. However, the proportion of patients with multiple fractures was increased as 23% of patients and 9% of controls had sustained multiple fractures.

We found lower trabecular density and decreased cortical thickness in the patients with APECED in line with previous studies on patients with adrenal insufficiency (12). Glucocorticoids act primarily on bone through actions on cells involved in bone remodeling (osteoblasts, osteocytes, and osteoclasts), and glucocorticoids are involved in the communication between various types of bone cells at different stages of differentiation. Reduced bone formation at trabecular bone sites and increased endocortical resorption are the most consistent pathological findings of glucocorticoid excess (12). In healthy children, higher adrenal glucocorticoid secretion within the normal hormonal range has been associated with smaller cortical area resulting from larger endosteal and lower periosteal circumference (13). Half of our patients had current daily hydrocortisone equivalent dose above 20 mg/day (0.32 mg/kg), although the presently recommended hydrocortisone dose is between 0.2-0.3 mg/kg (14). A recent prospective two-year study on adult patients with PAI demonstrated a significant increase in femoral neck and lumbar spine BMD Z-scores in the patients with cautious reduction in hydrocortisone equivalent dose (15). Although many of our patients received supraphysiological hydrocortisone equivalent doses, our study demonstrated vertebral fractures only in 7% of them by spinal radiographs, in comparison to a prevalence of 31% in patients with PAI in another study, when assessed with DXA at the same median age (16).

It is possible that hypoparathyroidism may protect the patients from glucocorticoid induced bone loss. Chronic deficiency of PTH leads to a reduction in bone remodeling and an associated increased BMD at all skeletal sites with highest BMD T/Z-scores at the lumbar spine (17–20). This was seen also in our cohort as 27% of the patients with hypoparathyroidism had high BMD, whereas none of the patients without hypoparathyroidism had high BMD. BMD is positively associated with the duration of hypoparathyroidism (18). The few studies have explored the overall risk of fractures in patients with hypoparathyroidism with contradictory results (20), but the largest studies have showed no significant difference in overall risk of fractures (21, 22). Several studies have demonstrated that both cancellous and cortical bone are abnormal in hypoparathyroidism. When assessing radius and tibia with high-resolution pQCT, cortical vBMD was higher, and cortical porosity and cortical thickness reduced in adult patients with hypoparathyroidim (19, 20).

When we evaluated parameters of bone metabolism with blood and urinary samples, the results reflected hypoparathyroidism when compared with healthy control subjects. Plasma ionized calcium was lower and phosphate was higher in adult patients with APECED. However, ALP, PINP and urinary INTP concentrations did not differ between the groups. The adult patients with APECED had higher urinary calcium secretion, and their daily calcium intake was higher. Only 55% of the patients used vitamin D_3_ supplements, and 24% of patients had 25(OH)D concentrations below 50 nmol/L. Altogether 82% of patients used either alfacalcidol or dihydrotachysterol medication for hypoparathyroidism. Thus, it is important to ascertain adequate vitamin D_3_ supplementation to maintain normal 25(OH)D concentration regardless of alfacalcidol medications.

Osteoporosis was found in 9% of the patients, and all of them had severe phenotype with multiple disease manifestations. In addition to PAI and hypoparathyroidism, patients with APECED may have hypogonadism, malabsorption, and growth hormone deficiency that may decrease the attained final height and peak bone mass. The adult patients with APECED were shorter and their BMI was lower than in controls subjects, which may explain partly the differences in bone architecture. Women with POI have increased risk of fractures and osteoporosis, but the current body of evidence of hormone replacement therapy on fracture risk and bone health is limited (23). In women with PAI who have not hypoparathyroidism, postmenopausal state increases the risk of bone loss, and glucocorticoid replacement therapy must be particularly carefully monitored (12). Adrenal insufficiency leads also to the lack of adrenal androgens that could be metabolized into more potent sex steroids. Adrenal androgen replacement with DHEA has been associated with reversal of bone loss only at femoral neck in a 12-month randomized placebo-controlled study on over 100 adult patients with PAI, and increased lean mass in female patients (24). Only 16% of our patients used DHEA replacement therapy. In addition of endocrinopathies and their treatments, malabsortion is a risk factor for compromised bone health. It may lead to osteomalacia that could not be determined by the methods of this study. Future studies should determine the magnitude of osteomalacia by careful histological and histomorphometric assessment of transiliac bone biopsies in patients with APECED.

Impaired bone structure was associated with more severe phenotype of APECED. Trabecular BMD and cortical thickness were lower in patients with 7 or more disease manifestations. In addition to endocrinopathies and malabsorption, the impaired immune functions may have direct effects on bone cells. In bone marrow, bone cells share the same micromilieu with immune cells. We know on the cellular level that osteoclasts utilize a defined set of molecules that play critical roles in immunomodulation including RANKL-RANK axis (25). In the mouse models, RANK-mediated signals are needed for the development of *Aire* expressing medullary thymic epithelial cells and RANK deficiency in thymus promotes the onset of autoimmunity (26). There have been no studies on the RANK-RANKL axis or bone cells in *Aire* knock-out rodent models or in the patients with APECED.

Although our cohort is one of the largest cohorts of APECED, the power of the study was limited. Especially in the subgroup analyses e.g., according to sex or menopausal status, the small number of subjects may have prevented us from detecting some meaningful differences between the groups. Due to cross-sectional nature of the study we were not able to define longitudinal changes in bone characteristics. The careful clinical follow-up data of the patients and our comprehensive evaluation of bone characteristics formed the solid basis of the study and increase the reliability of the results. Furthermore, our cohort is the largest cohort of patients with APECED that has thus far been studied for bone health, and this is the only study that has evaluated bone characteristics with pQCT. Due to the specific genetic background and established treatment guidelines in Finland, the results may not fully reflect bone health in the general APECED patient population. International collaborative efforts are needed to further explore skeletal consequences of APECED in different cohorts with various genetic constellations.

Patients with APECED need a comprehensive treatment with the special attention to bone health among all the other aspects of the disease. Reasonable glucocorticoid substitution, adequate treatments of hypogonadism and growth hormone deficiency together with the difficult handling of malabsorption can be the challenges when optimizing bone health during growth and puberty as well as when maintaining bone health during aging. The effects of altered osteoimmunology on the functions of bone cells and the magnitude of osteomalacia need to be elucidated in future studies on patients with APECED.

## Data Availability Statement

Restrictions apply to the availability of data generated or analyzed during this study to preserve patient confidentiality. The corresponding author will on request detail the restrictions and any conditions under which access to some data may be provided.

## Ethics Statement

The studies involving human participants were reviewed and approved by Research Ethics Committee of the Hospital District of Helsinki and Uusimaa. Written informed consent to participate in this study was provided by the participants' legal guardian/next of kin.

## Author Contributions

SL and OM contributed to conception and design of the study. SL, JB, ST-S, and MP were responsible for data collection. SL was responsible for data and statistical analysis, and wrote the first draft of the manuscript. ST-S, MP, and OM provided interpretation of the results. All authors contributed to manuscript revision, read, and approved the submitted version.

### Conflict of Interest

The authors declare that the research was conducted in the absence of any commercial or financial relationships that could be construed as a potential conflict of interest.

## Abbreviations

*AIRE*: autoimmune regulator gene
ALP: alkaline phosphatase
APECED: autoimmune polyendocrinopathy-candidiasis-ectodermal dystrophy
APS1: autoimmune polyglandular syndrome type 1
BMD: bone mineral density
25(OH)D: 25-hydroxy-vitamin D
INTP: collagen-1 aminoterminal telopeptide
PAI: primary adrenal insufficiency
PINP: procollagen type 1 aminoterminal propeptid
POI: primary ovarian insufficiency
pQCT: peripheral quantitative computed tomography
vBMD: volumetric bone mineral density.
